# Neuromuscular training and muscle strengthening in patients with patellofemoral pain syndrome: a protocol of randomized controlled trial

**DOI:** 10.1186/1471-2474-15-157

**Published:** 2014-05-16

**Authors:** Nayra Deise dos Anjos Rabelo, Bruna Lima, Amir Curcio dos Reis, André Serra Bley, Liu Chiao Yi, Thiago Yukio Fukuda, Leonardo Oliveira Pena Costa, Paulo Roberto Garcia Lucareli

**Affiliations:** 1 Department of Rehabilitation Science, Human Motion Analysis Laboratory, Universidade Nove de Julho, São Paulo, Brazil; 2 Universidade Federal de São Paulo, Campus Baixada Santista, Avenue Ana Costa, 95 - Santos/SP. CEP: 11060-000, São Paulo, SP, Brazil; 3 Irmandade da Santa Casa de Misericórdia, São Paulo, Brazil; 4 Masters and Doctoral Programs in Physical Therapy, Universidade Cidade de São Paulo, São Paulo, Brazil; 5 Musculoskeletal Division, The George Institute for Global Health, Sydney, Australia

**Keywords:** Biomechanics, Kinematics, Anterior knee pain, Hip, Knee, Neuromuscular

## Abstract

**Background:**

Patellofemoral pain syndrome (PFPS) is a common musculoskeletal condition, particularly among women. Patients with PFPS usually experience weakness in the gluteal muscles, as well as pain and impaired motor control during activities of daily living. Strengthening the hip muscles is an effective way of treating this disorder. Neuromuscular training has also been identified as a therapeutic tool, although the benefits of this intervention in patients with PFPS patients remain inconclusive.

**Design:**

This is a protocol of randomized controlled trial with a blind assessor. Thirty-four women with a clinical diagnosis of PFPS participated. These participants were allocated into two groups (experimental and control). The experimental group performed twelve sessions to strengthen the knee extensors, hip abductor and lateral rotator muscles in association with neuromuscular training of the trunk and lower extremities. The control group performed the same number of sessions to strengthen the muscles of the hip and knee. The primary outcome was functional capacity (Anterior Knee Pain Scale – AKPS) at 4 weeks. Pain intensity, muscle strength and kinematic changes were also measured during the step down test after four weeks of intervention. Follow up assessments were conducted after three and six months to assess functional capacity and pain. The effects of the treatment (i.e. between-group differences) were calculated using mixed linear models.

**Discussion:**

The present study was initiated on the 1st of April 2013 and is currently in progress. The results of this study may introduce another effective technique of conservative treatment and could guide physical therapists in the clinical decision-making process for women with PFPS.

**Trial registration:**

Current Controlled Trials NCT01804608.

## Background

Patellofemoral pain syndrome (PFPS) is a common musculoskeletal condition that is particularly prevalent among women [1,2]. Although described as multi-factorial, potential causes have commonly been associated with biomechanical disorders that are characterized by a deficit of dynamic stability in the trunk and lower limbs during weight-bearing activities, such as negotiating stairs, jumping and squatting [3-6].

Patients with PFPS usually exhibit a significant weakness of the lateral trunk flexors [7,8], as well as the hip abductor, lateral rotator muscles [9-11] and the knee extensors [12,13]. Exercise programs based on strengthening the quadriceps and gluteal muscles have been shown to decrease pain and improve motor function [14,15] and lower limb movement patterns [16].

It is common knowledge that these patients do not have normal control of lower limb movements [3,4,17] and exhibit deficient neuromuscular parameters, such as the activation time and electromyographic activity of the hip muscles [18]. It is also known that abnormalities of lower limb movement patterns during weight-bearing activities can directly affect referred pain [19].

Dynamic stability can be defined as the ability of the knee joint to maintain position (static stability) or intended trajectory (dynamic stability) after an internal or external disturbance [5]. The dynamic stability of the body, or any specific joint such as the knee, is contingent on neuromuscular control of the displacement of all contributing body segments during movement [20]. According to Zazulak et al. (2007), a deficit of neuromuscular control in the trunk can compromise the dynamic stability of the knee and lead to joint damage. Therefore, neuromuscular training, involving proprioceptive exercises related to disturbance and the correction of body sway, are indicated.

Salsich et al. (2012) also suggested that the correction of the dynamic alignment of the lower limbs could be an important component in the rehabilitation of these patients. However, until now, only three studies have investigated this aspect, and two of them only assessed the influence of correcting gait mechanics [21,22]. The third study compared a program of hip muscle strengthening exercises, associated with training to control the movement of the trunk and lower limbs, with a program that only focused on strengthening the quadriceps [23].

It is known that patients with PFPS exhibit abnormalities in the mechanics and dynamic control of lower limbs. However, very few studies have used neuromuscular training as a treatment strategy and there is insufficient evidence about the influence of this intervention in terms of the clinical and biomechanical aspects of these patients.

The aim of the present study was to compare the effects of a program of neuromuscular training of the trunk and lower limbs, associated with strength training of the hip and knee muscles of women with PFPS, with a program that only involved strengthening of these same muscles in relation to functional capacity. Our hypothesis is that the group submitted to the program that associated strength and neuromuscular training would exhibit better results than the group that only received the strength training.

## Methods

### Study design

**Figure 1:**
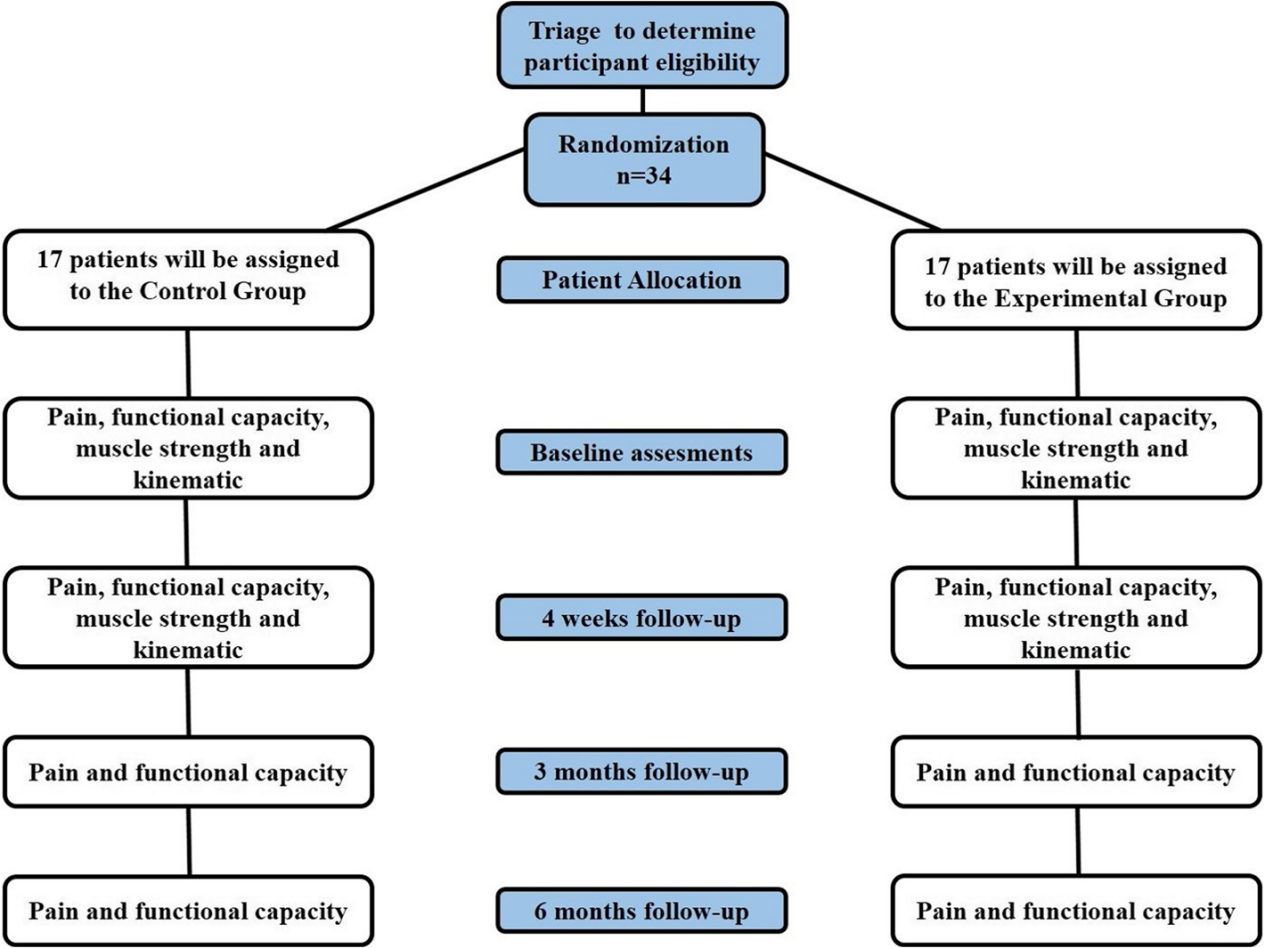
Fluxogram of the study design.

The present study was a randomized controlled trial with a blinded assessor (Figure 1). This trial was approved by the Human Rights Ethics Committee of the *Universidade Nove de Julho* (UNINOVE) under protocol number 124.075. This study was registered on ClinicalTrials.gov (trial registration number NCT01804608) and did not receive any external funding. This trial started recruiting patients on the 1st of April 2013 and data collection is likely to finish by July 2014.

After 4 weeks, the intervention was performed to assess the functional capacity, pain intensity, muscle strength and kinematics of the trunk and lower limbs. Functional capacity and pain intensity were reassessed three and six months after randomization.

### Participants, therapists, centers

Thirty-four sedentary women were recruited for the present study. These participants had to have anterior knee pain for at least three months during at least two of the following activities: remaining seated for a prolonged time; going up or down stairs; squatting; running and jumping [24,25]. Participants aged between 18 and 30 years were included. Individuals with a history of surgery in the lower limb, chronic instability of the knee, disorders associated with meniscal and/or ligamentous injuries, as well as cardiac or locomotor disorders that could affect the assessment and treatment, were excluded from the present study, as were those with a discrepancy of more than one centimeter in leg length.

After signing a consent form to participate in the present study, the subjects were randomized to receive one of the treatment options. These patients were treated by two physical therapists that had been trained to apply the interventions and have experience in musculoskeletal physical therapy. Each patient was treated by a single therapist, who was not involved in the assessment of the patients. The research was conducted in the physical therapy clinic of the *Universidade Nove de Julho* (UNINOVE) in São Paulo, Brazil.

### Procedure

The randomization schedule was developed by an investigator who was not involved in the recruitment, treatment or assessment of patients. The randomization codes were generated using the RAND function of Microsoft Excel for Windows. Opaque sealed and sequentially numbered envelopes were used in order to conceal the allocation. The therapist that carried out the treatment opened the envelopes with the random codes.

The professional responsible for the assessments was blinded for the treatment allocation. However, the patients were informed that they would receive one of the two forms of treatment, and therefore cannot be considered as blinded. Due to the nature of the interventions, it was not possible to blind the physiotherapists.

### Intervention/control

Thirty-four patients were randomly allocated into two groups with two different treatment programs:

Control Group (CG) – submitted to strengthening exercises of the knee extensors, hip lateral rotators and abductors.

Experimental Group (EG) – submitted to the same program of strengthening as the CG on stable and unstable ground, with coordination through verbal commands to improve the dynamic motor control of the lower limbs.

Subjects from both groups performed three treatment sessions per week, for a period of four weeks, totaling 12 sessions of 60 minutes. In each session, 10 minutes of warming up on a treadmill or exercise bike was performed prior to the intervention. During the study period, the volunteers were asked not to seek any other type of treatment for anterior knee pain and to maintain their regular daily activities. They were monitored during the sessions.

**Figure 2:**
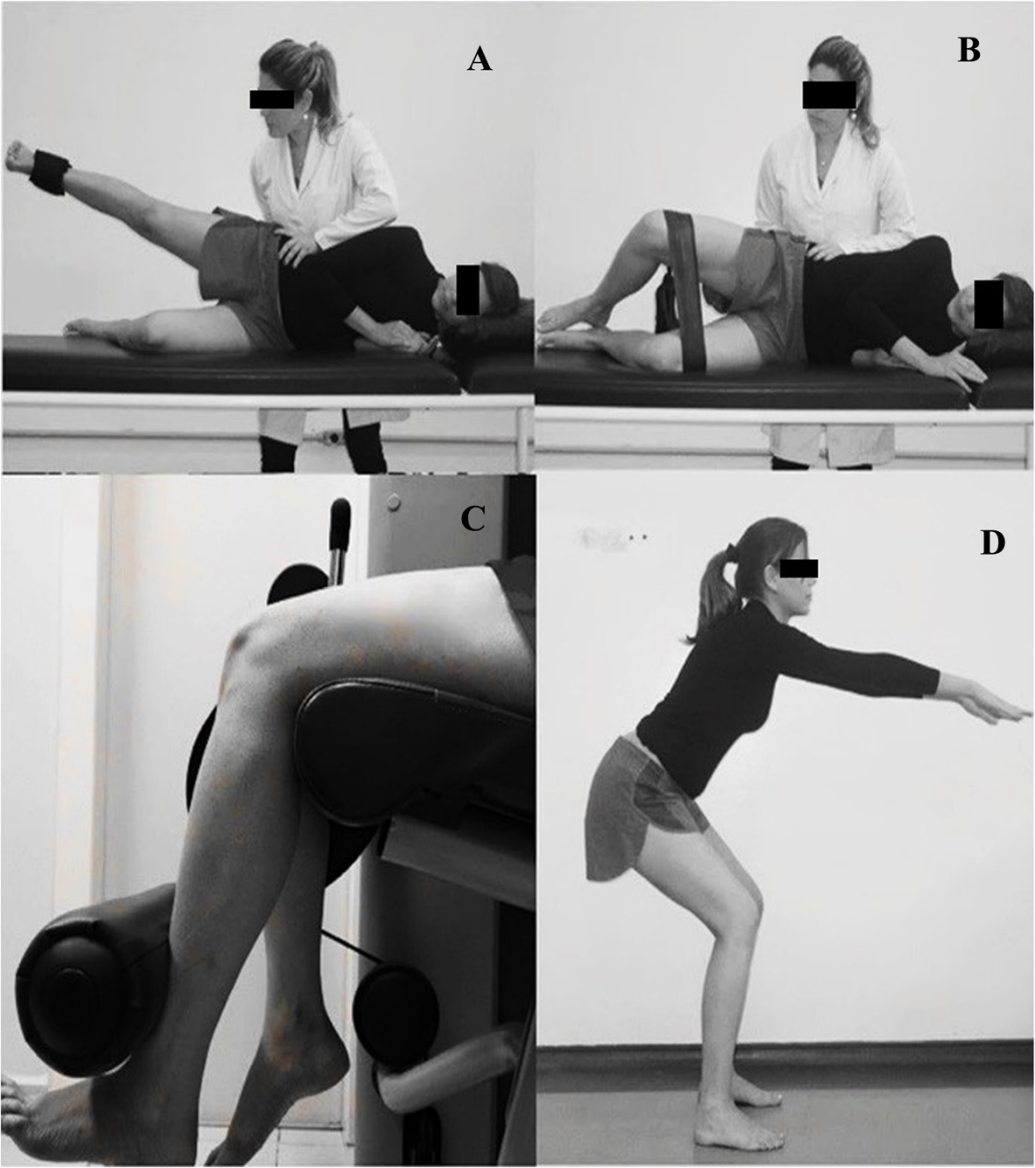
**Strengthening of the hip and knee muscles in the first week of treatment. A)** Straight Leg Raise (SLR) with slight hip extension. Physiotherapist stabilizes the pelvis to avoid compensatory movement; **B)** Abduction and lateral rotation at 30° of the hip flexion (clam) with resistance elastic around the knee. During the execution of the movement the therapist stabilizes the patient’s pelvis; **C)** Quadriceps strengthening without weight bearing. Initial position 90° and final position 45° of the knee flexion, such as safe angulation for the patellofemoral joint; **D)** Squat preventing the knee exceeds the midfoot.

The literature has already tested the effectiveness of strengthening the knee and hip muscles in PFPS patients with positive results [14,16,26]. This treatment method has had favorable results in terms of pain, motor function and movement patterns. One of the reasons for this success is that these patients exhibited abnormalities in the dynamic alignment of the patella and significantly reduced strength in the hip muscles (lateral rotators, abductors and extensors) [9,11]. Therefore, the subjects in the control group in the present study were submitted to the following strengthening exercises:

Side lying hip abduction (1st-4th week): The patient was in lateral decubitus and started from a position of complete knee extension, with the hip in a neutral position. They were asked to slowly abduct the hip of the higher limb until they reached 30°, holding it in a neutral position on the transverse plane. The therapist was positioned behind the patient to avoid the pelvis moving upwards while performing the exercise. Resistance was applied in the distal third of the leg (Figure 2A).

Side lying clam exercise (1st-4th week): The patient was in lateral decubitus with the feet together, the hips and knees flexed at approximately 45° and an elastic band tied around the knees (Figure 2B). The patient was told to hold the feet together and to lift the knee, which occurred through abduction and lateral rotation of the hip. The therapist was positioned behind the patient to avoid the trunk or pelvis moving posteriorly while performing the exercise.

Knee extension (1st-4th week): The patient was in a chair at 90° of knee flexion and the hip performed extension starting from 90° and finishing at 45° of knee flexion. The exercise was performed unilaterally and resistance was applied on the ventral side of the distal third of the leg (Figure 2C).

Squat (1st-4th week): The patient stood with the hips in a neutral position, the knees extended and the feet parallel and shoulder-width apart. The patient was asked to perform a squat with the leg remaining perpendicular to the ground, until they reached 30° of knee flexion. The patient was asked to squat by flexing the hip and the trunk more (Figure 2D).

**Figure 3:**
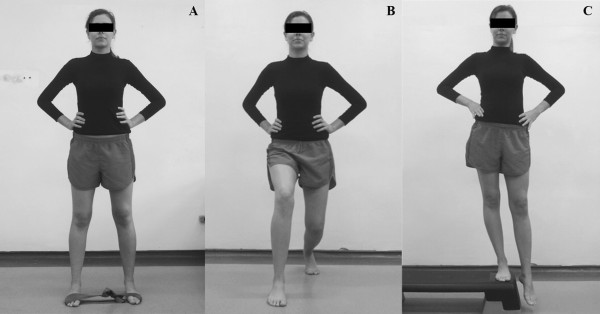
**Strengthening of the hip and knee muscles added in the second (A) and third week (B and C). A)** Lateral walk with elastic resistance around the forefoot, **B)** Forward lunge **C)** Strengthening the hip abductors with weight bearing (Trendelenburg).

Lateral band walks (2nd-4th weeks): The patient stood with the knees and hips at 30° flexion, feet parallel, hands on hips and an elastic band around the forefoot. The patient was asked to walk sideways, performing active abduction with one of the limbs and slowly abducting the other. Shoulder distance was used as a reference for hip abduction. The patient was instructed not to perform lateral compensation movements of the trunk or elevation of the hip (Figure 3A).

Forward lunge (3rd-4th weeks): The patient stood with the feet parallel to start and then stepped forward with the hands on their hips and the trunk in a vertical position. They were asked to squat so that the hind leg remained perpendicular to the ground (Figure 3B).

Eccentric adduction of the hip with weight-bearing (3rd-4th weeks): The patient stood with one of the lower limbs on a step and the other suspended at the same level immediately beside it, with hands on hips and the trunk and pelvis in a neutral position. They were asked to perform hip adduction by trying to touch the ground with the foot of the limb that was suspended and then returning to the starting position without making a compensatory movement of pelvic elevation contralateral to the weight-bearing limb. Resistance was applied to the distal third of the leg (Figure 3C).

The load during training was standardized as 70% of a maximum repetition [27], which is the maximum load that a person can support to complete a repetition of an exercise without pain. This maximum load was assessed during the first session and revised on a weekly basis for necessary adjustments. Exercises using elastic resistance were standardized for the maximum load that each patient could support while completing 10 repetitions of the exercise. This resistance was also assessed on a weekly basis for adaptations. These criteria were based on the protocol described by Fukuda et al. (2010). The patients performed 3 sets of each exercise (one-minute intervals between sets), with 15 repetitions. Resistance was increased as soon as the exercise became easy to execute.

**Figure 4:**
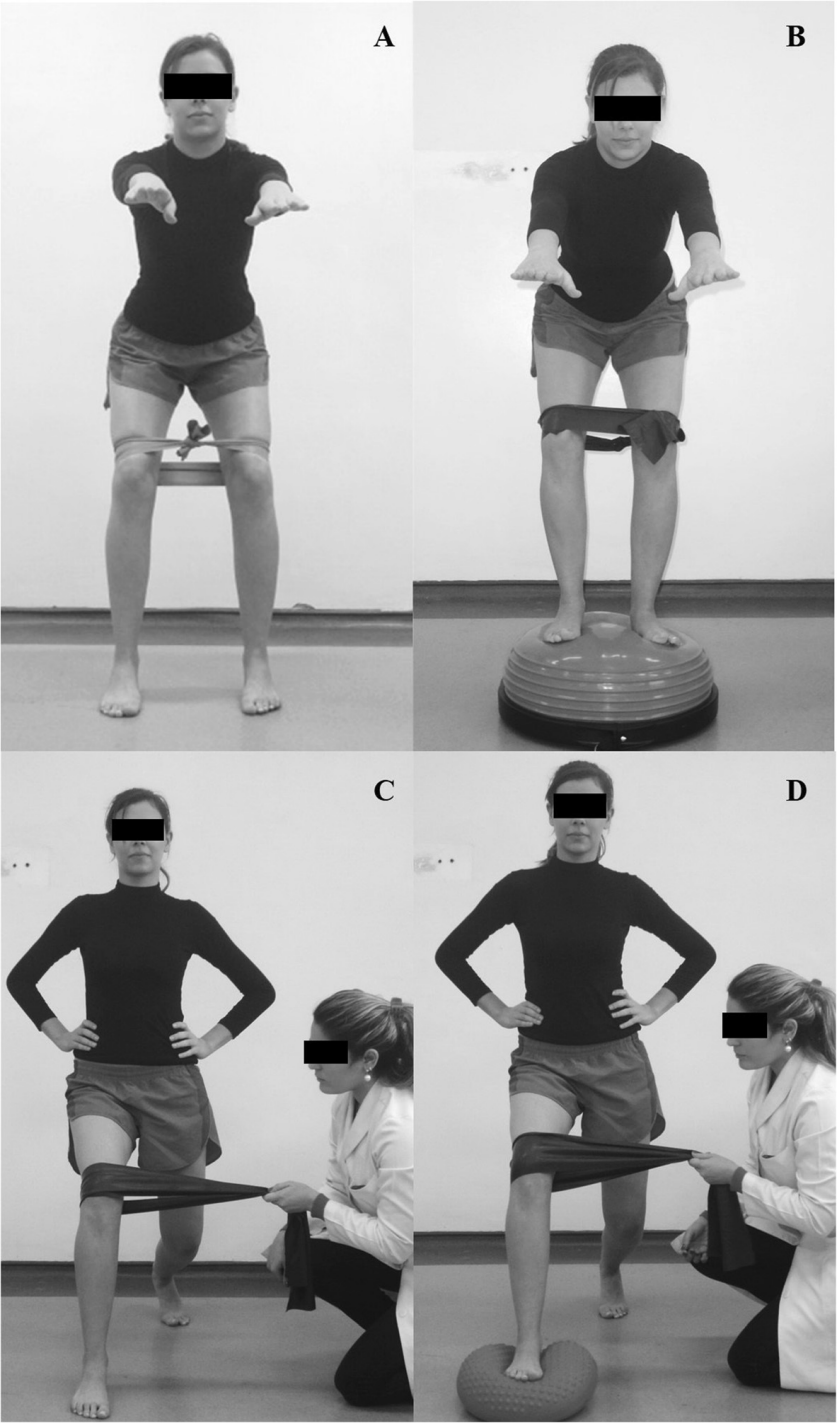
**Double leg neuromuscular training associated with strengthening exercises. A** and **B)** Squat with elastic resistance around the knees stimulating the constant activation of the hip abductors and lateral rotators durinig task execution. Respectively stable and unstable terrain; **C** and **D)** Modified forward lunge with elastic around the knee that is ahead for constant muscle activation abductors and lateral rotators of the hip and training of motor control during the execution of the activity. Respectively stable and unstable terrain.

As well as poor dynamic alignment of the patella and weak hip muscles, PFPS patients exhibit abnormal trunk and lower limb movement patterns during weight-bearing and one-foot activities [11,28,29]. Thus, patients in the experimental group of the present study were submitted to the same protocol as the CG, with added proprioceptive stimuli and the following exercises:

Modified squat (1st-4th week): In the first and second weeks, the patient performed the squat exercise as described for the CG, although they had an elastic band around their knees. This band stimulated adduction of the femur but the patient was instructed to withstand this resistance and always keep the knees in the direction of the hips throughout the exercise (Figure 4A). In the third and fourth weeks, the patient performed this squat with elastic resistance on an unstable surface (Figure 4B).

Modified forward lunge (3rd-4th weeks): The patient performed the exercise as described for the CG but the therapist positioned an elastic band around the knee of the limb that was positioned in front and stimulated adduction. However, the patient was told to withstand this resistance and always keep the knee in the direction of the hips (Figure 4C).

**Figure 5:**
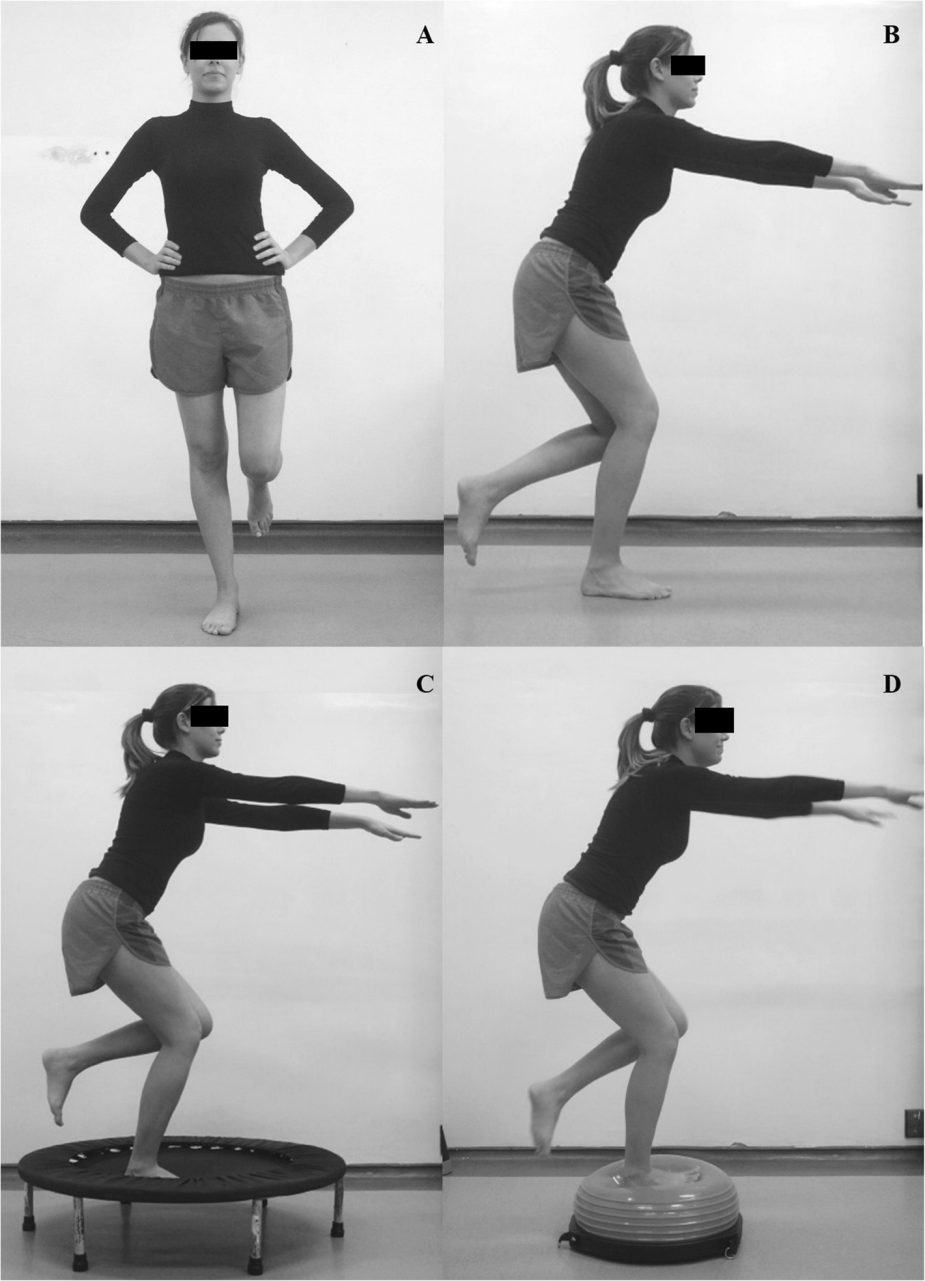
**One leg neuromuscular training associated with strengthening exercises. A)** One-leg balance with knee extension, on stable terrain; **B** and **C)** One-leg balance at 30° of knee flexion, on stable and unstable terrain, respectively; **D)** Unipodal squat. These activities should keep the pelvis balanced and avoid excessive pronation of the foot.

One-leg balance with extended knee (1st week): The patient stood on one foot with the knee extended and the pelvis and trunk in a neutral position. They held this position, keeping their balance and avoiding compensatory movements such as rotation and inclination of the trunk and pelvis or pronation of the support foot. In the first week, the patient performed this exercise on a flat surface (Figure 5A). They performed three sets of 20 seconds for each limb.

One-leg balance with knee flexion (2nd-3rd weeks): The patient stood on one foot with the knee at 30° flexion, keeping the leg of the support limb perpendicular to the ground and the hip and trunk slightly flexed. The patient was asked to hold this position, keeping their balance and avoiding compensatory movements such as rotation and inclination of the trunk and pelvis, adduction and medial rotation of the hip or pronation of the support foot. In the second week, the patient performed this exercise on a flat surface (Figure 5B) whereas in the third week, it was done on an unstable surface (Figure 5C).

One Leg Squat (4th week): The patient stood on one foot with the knee extended and the hip, pelvis and trunk in a neutral position. They were asked to squat slowly until the knee reached 30° of flexion. The patient was then asked to squat while flexing the hip and trunk more, keeping the leg of the support limb perpendicular to the ground and avoiding compensatory movements such as rotation and inclination of the trunk and pelvis, adduction and medial rotation of the hip or pronation of the foot (Figure 5D).

All patients allocated to this group received detailed explanations of the movement disorder that they exhibited when they performed weight-bearing activities (ipsilateral trunk lean and contralateral pelvic drop, hip adduction and medial rotation and foot pronation) [11]. They were also informed how to correct this abnormality while performing the exercises and were verbally stimulated by the therapist. All exercises were performed in front of a mirror in order to provide visual feedback. The patients performed three sets of each exercise (one-minute intervals between sets), with 15 repetitions. Similar to the CG, the resistance was increased at the moment when the exercise became easy to perform. The correction of body sway exercises was also conducted in sets of three and held for the following period of time: 20 seconds in the first week; 30 seconds in the second week and 40 seconds in the third week.

### Outcome measures

Four outcome measures were used before and after the interventions: a) Anterior knee pain intensity during activities of daily living was measured by the Anterior Knee Pain Scale – AKPS [30]; b) Pain Intensity was measured by the Numerical Pain Rating Scale - NPRS [30]; c) Maximum isometric muscle force of the knee and hip muscles was measured by the manual dynamometer (Lafayette Instrument Company, Lafayette, IN); d) Kinematic assessment of the trunk and lower limbs during the step down test was performed by the Vicon® movement analysis system.

The above-mentioned scales, which have been translated to Portuguese and cross-culturally adapted for the Brazilian population, are often used to measure pain and functionality in PFPS patients [14,30,31]. The dynamometer is a reliable tool both for inter and intra-assessments of the strength of the knee and hip muscles in women [32,33]. Kinematic analysis is a reliable and commonly used assessment method to quantify lower limb movements [11].

### Primary outcomes

The primary outcome was functional capacity, measured after treatment (4 weeks) using the Anterior Knee Pain Scale – AKPS [30].

### Secondary outcomes

The secondary outcomes were pain intensity, muscle strength of the hip abductors and lateral rotators, extensors of the knee and three-dimensional kinematics of the trunk, pelvis, hip, knee and ankle during the step down test, measured after treatment (4 weeks). In addition, functional capacity and pain intensity were assessed three and six months after randomization.

The description of each of these measurements is described below.

**Anterior Knee Pain Scale – AKPS**: This is a specific questionnaire for anterior knee pain, composed of 13 items that are separated in categories that involve different levels of knee function. Each item was answered and the total result was added to a global index with a maximum score of 100 points, which represents “no deficit”, and a minimum score of zero, representing “the highest possible deficit” [30,34].

**Numerical Pain Rating Scale - NPRS**: This scale measures pain intensity. The score ranges from 0 to 10 points, with 0 representing a “complete absence of pain” and 10 representing the “worst possible pain” [30]. The patients were asked to classify their pain intensity based on the last seven days and while performing the step down test.

**Manual Dynamometer (Lafayette Instrument Company, Lafayette, IN):** This tool is used to quantify muscle strength during maximum voluntary isometric contraction, with adequate reliability in both inter and intra-assessments [32,33]. The abductors, lateral rotators and extensors of the hip and the knee extensors were assessed as described by Bolgla et al. (2011).

**Kinematic Analysis of the Step Down Test:** The Vicon-Nexus® system was used, which involves 8 infrared cameras capable of 250 frames per second (fps) and a resolution of 1MP 1024×1024. This system captured the movements of the reflective markers that were placed on specific anatomical points of the patient’s skin, based on the Vicon® Plug in Gait model [35,36]. All of the cameras were connected to a computer dedicated to treatment of the video signal, which had different functions: timing circuit/control; coordinate generating circuit and interface circuit of the cameras. Once stored in the video memory, the data were transferred to a second general purpose computer. Vicon-Nexus® software was used to process and reconstruct 3D images of the markers through a biomechanical model and a number of mathematical algorithms.

Static calibration of the system was performed to determine the laboratory reference coordinates (X, Y and Z). Subsequently, dynamic calibration was carried out scanning the volume of interest with a metal rod in the shape of a “T” containing five markers. All of the participants wore shorts to enable the placement and reading of the markers. Their skin was cleaned with cotton and 70% alcohol to improve the fixation of the markers. Twenty-one retro-reflective double-sided spherical markers (14 mm diameter) were fixed (3 M®) to specific anatomical points, which served as a reference for the motion analysis capture system.

The markers were placed in the following locations: on the spinous process of the 7th cervical vertebra; on the spinous process of the 7th thoracic vertebra; on the jugular notch; xiphoid process; anywhere over the right scapula; on the left and right acromioclavicular joint; two anterior and posterior superior iliac spines; in the lateral femoral epicondyles; on the lateral aspect of the thighs; on the lateral malleolus; over the second metatarsals and on the calcaneus. This set of markers was placed following the conventional gait model [35,36].

This set of markers was based on the Helen Hays model that was used to estimate the position of the articular centers, as well as to calculate the three-dimensional kinematics of the pelvis, hip, knee and ankle joints [35,36]. The same experienced examiner positioned the markers in all conditions.

After the capture of the coordinates of the markers, they were named and saved in C3D format. In order to reconstruct the three-dimensional biomechanical model, the coordinates of the markers fixed to the trunk, pelvis, thigh, leg and foot were imported and processed in Vicon Nexus® software. The kinematic data were filtered using a fourth-order zero-lag Butterworth 8-Hz low-pass filter. Joint kinematics were calculated using a joint coordinate system approach [35,36] and were reported relative to a static standing trial in order to quantify the movement of one segment in relation to another or of one segment relative to the laboratory.

The step down test was selected for the three-dimensional analysis. This functional test has often been described in the literature as an assessment method for the quality of lower limb movements [37-40].

The patient was positioned on a step (18 cm high and 30 cm wide and deep) with the limb to be tested close to the edge and the non-tested limb suspended (both starting from the same position). The volunteer was asked to squat slowly (for two seconds) until the heel of the non-tested limb touched the ground and then return immediately to the starting position for two seconds (the exercise was repeated until three consecutive squats were completed). The patient performed the activity three times with an interval of one to two minutes between them, or until they felt ready to do the test again. Before execution, the examiner demonstrated the exercise and gave verbal instructions. Once the participant had confirmed that they understood what they were going to do, they practiced the activity once to become familiar with the movement.

The height of the step was regulated so as to adjust to a 60° angle of knee flexion in the support leg at the moment that the contralateral foot touched the ground. The participant was asked to squat until they reached this knee angle, which was measured by a goniometer. If they achieved the 60° angle and the heel of the non-tested side had still not touched the ground, this distance was adjusted by placing blocks of ethylene-vinyl-acetate (E.V.A.) on the ground. If the heel touched the ground before the required angle was attained, the height of the step was adjusted.

### Data analysis

The normality of the data will be confirmed by a visual inspection of the histograms. A logarithmic transformation [log(x + 1)] for the complete set of data will be used for the variables of asymmetric distribution. In cases where non-parametric distribution persists, all comparisons will be performed with the raw data (without transformation), using the non-parametric tests. The effects of the interventions (between-group differences) will be calculated using linear mixed models by using interaction terms (group allocation versus time), which is equivalent to the between-group differences. The within-group differences will be calculated using repeated measures ANOVA. P-values < 0.05 will be considered as statistically significant. The data will be double checked before analysis. The Statistical Package for the Social Sciences (SPSS) software was used for all analysis.

The clinical relevance of the results will be confirmed by calculating the effect size (Cohen *d*) of the significant differences found between the assessments. The following effects will be considered: 0.00-0.49 small; 0.50-0.79 medium and above 0.80 great (Cohen, 1988).

The calculation of the sample size was performed to detect a difference of 8 points on the Anterior Knee Pain Scale (AKPS) (with a standard deviation estimated at 7.5 points). The alpha level was 0.05 and the statistical power was 80%. Therefore, the sample size required (per group) was 15. We decided to increase the sample to compensate for the loss of patients or to compensate for abnormally distributed data. Each group was increased to 17 (i.e. total sample size of 34 participants).

## Discussion

Although PFPS treatment with an emphasis on strengthening the hip muscles is well established in the literature and has demonstrated satisfactory results for the clinical problem and kinematic abnormalities [14,16,26,31,38], the role of neuromuscular training with an emphasis on controlling lower limb movements remains relatively unclear [19,25,41].

Mascal, Landel and Powers (2003), in a report of two cases, demonstrated an improvement in pain, kinematic changes in the lower limbs and an improvement in functional capacity during the step down test in PFPS patients who participated in a protocol of gluteal muscle strengthening and neuromuscular training. However, since it was a case-series, the results cannot be considered as robust enough for implementation in clinical practice. Recently, Salsish et al. (2012) demonstrated that voluntary alteration of lower limb movement patterns could directly affect referred pain in PFPS patients while squatting. However, the effect observed in the present study was only recorded during the execution of the movement. There is a lack of studies in the literature that adopt a neuromuscular training program to correct or reduce existing biomechanical abnormalities in patients with patellofemoral disorders, as well as studies that analyze the effect of this therapeutic method in association with other techniques that are already known. Therefore, the conclusions of the present study could provide significant information about neuromuscular training in patients with PFPS.

A greater understanding of the effects of sensory motor training on the clinical and biomechanical aspects of PFPS patients could clarify the situation and consolidate the concept of another potentially effective conservative treatment, as well as guiding physiotherapists in the clinical decision-making process for PFPS patients.

## Abbreviations

AKPS: Anterior knee pain scale; CG: Control group; EG: Experimental group; FIQ: Functional index questionnaire; NPRS: Numerical pain rating scale; PFPS: Patellofemoral pain syndrome.

## Competing interests

The authors declare that they have no competing interests.

## Authors’ contributions

NR, TF, LC, and PL, AC and AB contributed to the development and, concept and the design of the protocol. NR, TF, LC, PL, BL and LY participated of the protocol design. NR has drafted the manuscript with critical input from all other authors who have read and approved the final manuscript.

## Pre-publication history

The pre-publication history for this paper can be accessed here:

http://www.biomedcentral.com/1471-2474/15/157/prepub
